# Yields and Constituents of Essential Oil from Cones of *Pinaceae* spp. Natively Grown in Turkey

**DOI:** 10.3390/molecules15085797

**Published:** 2010-08-24

**Authors:** Ibrahim Tumen, Harzemsah Hafizoglu, Ayben Kilic, Ilhami Emrah Dönmez, Huseyin Sivrikaya, Markku Reunanen

**Affiliations:** 1 Faculty of Forestry, Bartin University, 74100 Bartin, Turkey; 2 Department of Wood and Paper Chemistry, Åbo Akademi University, 20500 Turku, Finland

**Keywords:** essential oil, terpenes, Pinaceae, fir, cones, GC/MS

## Abstract

In this study, the yields and composition of essential oils obtained from the cones of Pinaceae family species natively grown in Turkey were investigated. Essential oils were obtained by hydrodistillation. Oil yields were 0.13-0.48 mL/100 g in pine cones, 0.42-0.59 mL/100g in fir, 0.36 mL/100g in spruce and 0.37 mL/100g in cedar. While α-pinene (47.1-14.8%) was the main constituent of *P*. *slyvestris, P. nigra* and *P. halepensis*, limonene (62.8%) in *P. pinea* and β-pinene (39.6%) in *P. brutia* were found in higher amounts. Like in *P. pinea*, limonene was the main compound in *Cedrus libani* (22.7%). In fir species the major compounds were α-pinene (70.6-53.0%) and β-pinene (10.9-8.2%). Contrary to other species β-pinene (32.7%) was found as a major compound in *Picea orientalis*.

## 1. Introduction

Essential oils are used in many industrial fields for the perfuming and flavouring of various products [1,2]. Essential oils are natural, complex, multi-component systems composed mainly of terpenes, in addition to some other non-terpene components. Several techniques can be used to extract essential oils from different parts of the aromatic plant, including water or steam distillation, solvent extraction, expression under pressure, supercritical fluid and subcritical water extractions [3].

Essential oils from aromatic and medicinal plants have been known since antiquity to possess biological activity, most notably antibacterial, antifungal and antioxidant properties. With growing interest in the use of essential oils in both the food and pharmaceutical industries, the systematic examination of plant extracts for these properties has become increasingly important. The terpene composition of seed cone oleoresin has been reported, and headspace techniques were developed to isolate volatile compounds from plant odour compounds in order to determine the composition of the host odour, which is attractive to insects [4,5,6,7,8].

Turkey, because of its geographical position at the crossing region of temperate continental and Mediterranean climates, is rich in coniferous woods that grow in different regions of the country, occupying about half of the county’s total forest area [9,10]. 

Five pine species are recorded in Turkey (*Pinus brutia, Pinus nigra, Pinus sylvestris, Pinus pinea, Pinus halepensis*) and three of them (*P. brutia, P. nigra, P. sylvestris*) are commercially utilized. Previous studies on Pinus species in Turkey were mainly focused on improving the yield of turpentine production. Pine oils are widely used as fragrances in cosmetics, as flavoring additives for food and beverages, as scenting agents in a variety of household products, and intermediates in the synthesis of other perfume chemicals [11,12].

Fir species exhibit parallel variation in indumentum characteristics and in the presence or absence of resinous buds. These features are well correlated with their geographical distribution [13]. Fir species are represented in Turkey by *Abies nordmanniana* (Stev.) Spach., *Abies bornmulleriana* Mattf., *Abies equi-trojani* (Asch.&Sint. ex Boiss.) Mattf. and *Abies cilicica* (Ant. et Kotschy) Carr. *A. bornmulleriana*, *A. equi-trojani* and *A. cilicica* subsp. *isaurica* are also endemic plants in Turkey [14,15]. *A. nordmanniana*, *A. bornmulleriana*, *A. equi trojani* are distributed in northern Turkey and *A. cilicica* (Ant. et Kotschy) Carr. is distributed in southern Turkey [13,16,17,18].

Cones of some coniferous species find uses in industry [19]. Essential oil constituents of the cones of the family Pinaceae are poorly known, although there have been some studies on the antioxidant activity, terpenoids, steroids, anti-HIV activity, procyanidins, *etc.* of all the Pinaceae cones [20,21,22]. 

## 2. Results and Discussion

**Figure 1:**
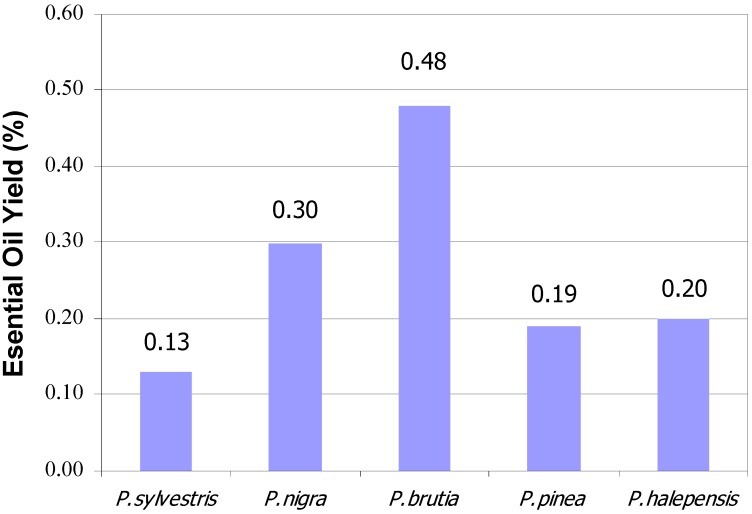
Essential Oil Yields of Pine Species from Turkey (%).

**Figure 2:**
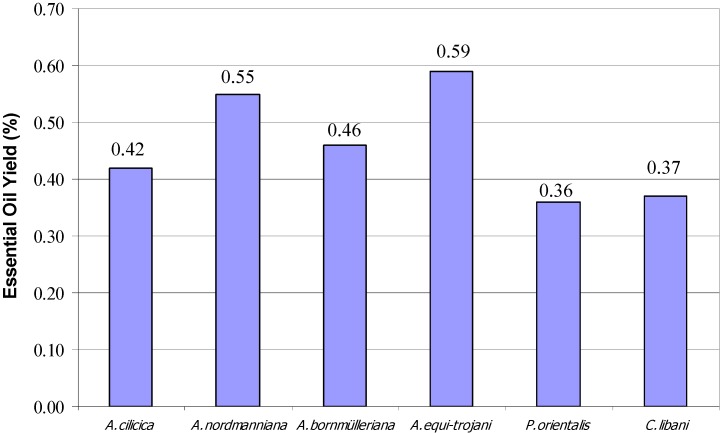
Essential Oil Yields of Fir Spruce and Cedar species from Turkey (%).

Oil yields of pine species are given in Figure 1 and those of fir, spruce and cedar species given in Figure 2. Evidently, the highest oil content (0.48%) was found in *P. brutia* and the lowest (0.13%) in *P. sylvestris* among the pine species. The essential oil compounds of pine cones are given in Table 1 and those of fir, spruce and cedar in Table 2. As can be seen from these tables, the main compounds were as follows: α-pinene, β-pinene, β-myrcene, Δ^3^-carene, limonene and β-caryophyllene. α-Pinene was the major compound in the cones of *Pinaceae* family. This compound was also found to account for more than 50% of the contents in the fir species too. α-Pinene was also identified as a major compound in *P. nigra* (45.36%) and *P. halepensis* (47.09%). Except for *P. brutia* and *P. orientalis*,β-pinene was found to be the second most important component in all cones. In the *P. brutia* (39.56%) and *P. orientalis* (32.67%) samples this compound was the most abundant compound. Limonene was the dominant component in *P. pinea* (69.54%, combined with β-phellandrene) and in *C.libani* (17.71%). This terpene is used as an antimicrobial inhibitor in the food industry. Although β-caryophyllene, an important sesquiterpene, was found to be less than 1% in the Abies species, the amount of this compound was more than 10% in *P. halepensis* (11.22%). 

As it can be seen in Figure 2, the highest essential oil yield of cones was found in *A. equ-i trojani* with 0.59% and the lowest was in *P. orientalis* at 0.36%. Among the *Pinaceae* family the highest essential oil yield was observed in *A. equi trojani* and the lowest was determined in *P. sylvestris*.

**Figure 3:**
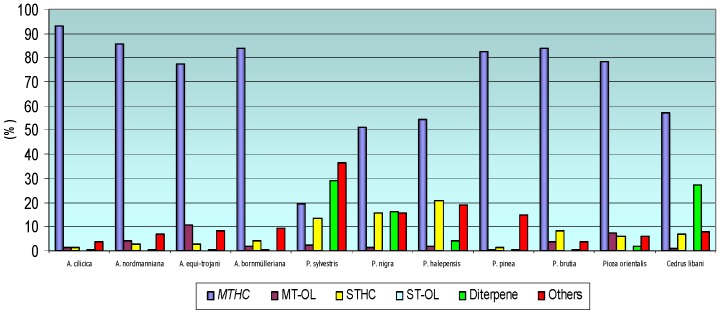
Terpene groups of Pinaceae Family Native Grown in Turkey (%).

Terpene groups in the essential oils of *Pinaceae* cones were investigated. The terpenes were grouped into monoterpene hydrocarbons, monoterpene alcohols, sesquiterpene hydrocarbons, sesquiterpene alcohols and diterpenes. Those terpene groups and their amounts in different cones are given in Table 3 and Figure 3. Monoterpene hydrocarbons was found to be at the highest level in *A. cilicica* (93.14%), the highest level of monoterpene alcohols was found in *A. equi-trojani* (10.70%), sesquiterpene hydrocarbons were highest in *P. halepensis* (20.82%), diterpenes were the highest in *P. sylvestris* at 28.94%.

**Table 1:** Percent (w/w %) composition^1)^ of components in Pines.

Nr	RI	Compounds^2)^	*P.* *pinea*	*P. brutia*	*P. sylvestris*	*P.* *nigra*	*P. halepensis*
1	925	tricyclene	0.02	0.04	0.05	0.08	0.16
2	936	α-Pinene	17.90	30.91	14.76	45.36	47.09
3	949	camphene	0.32	0.64	0.60	1.04	0.88
4	955	2,4 (10)-Thujadiene	0.19	0.15	0.62	0.39	0.60
5	978	β-Pinene	1.70	39.56	1.78	1.50	2.75
6	989	β-Myrcene	0.79	1.13	0.17	0.16	6.25
7	1010	Δ^3^-carene	-	7.80	-	-	1.72
8	1021	p-cymene	0.29	0.37	0.47	0.30	0.42
9	1026	limonene + beta-phellandrene	69.54	2.05	0.48	1.88	0.79
10	1087	α-terpinolene	0.04	0.48	0.04	0.03	0.09
11	1088	p-cymenene	0.13	0.14	0.23	0.16	0.21
12	1123	α-campholene aldehyde	0.27	0.13	0.87	0.47	0.45
13	1135	trans-pinocarveol	0.47	1.38	0.91	0.49	0.88
14	1138	cis-verbenol	0.06	0.03	0.02	0.02	0.24
15	1141	trans-verbenol	0.25	0.07	0.05	0.07	0.71
16	1145	p-mentha-1,5-dien-8-ol^3)^	0.08	0.11	0.49	0.26	0.38
17	1156	pinocarvone	0.10	0.42	0.22	0.15	0.27
18	1162	borneol	-	0.10	0.03	0.07	0.11
19	1165	p-mentha-1,5-dien-8-ol	0.18	0.24	1.07	0.42	1.03
20	1177	4-terpineol	0.08	0.26	0.01	0.03	0.08
21	1190	α-terpineol	0.14	0.93	0.41	0.77	0.30
22	1194	myrtenal + myrtenol	0.47	1.30	1.22	0.58	0.91
23	1206	verbenone	0.08	0.08	0.59	0.12	0.22
24	1218	trans-carveol	0.64	0.04	0.04	0.09	0.12
25	1286	bornyl acetate	0.26	0.22	0.02	0.69	0.64
26	1348	α-longipinene	0.18	-	-	-	-
27	1374	α-copaene	-	0.06	0.06	0.24	0.34
28	1403	longifolene	0.52	0.06	1.16	0.34	-
29	1420	β-caryophyllene	0.73	5.01	2.87	6.73	11.22
30	1453	α-humulene	0.10	1.25	0.61	1.48	2.65
31	1484	germacrene-D	-	0.38	0.01	0.06	0.02
32	1502	α-muurolene	-	0.06	0.02	0.11	0.31
33	1514	gamma-cadinene	-	0.04	0.01	0.09	0.25
34	1525	trans-calamenene + Δ-cadinene	-	0.13	0.01	0.28	0.32
35	1578	caryophyllene oxide	0.33	1.61	12.58	8.05	7.47
36	1607	humulene epoxide	-	0.23	1.48	1.07	1.11
37	1960	19-norabieta-8,11,13-triene^3)^	0.02	0.03	4.75	0.70	0.37
38	1974	isopimaradiene^3)^	-	-	-	0.26	0.81
39	1974	manoyl oxide	-	0.33	-	-	-
40	1987	norabieta-4(18),8,11,13-tetraene^3)^	0.02	0.03	4.59	0.50	0.17
41	2002	manoyl oxide	0.25	-	-	-	-
42	2005	palustradiene^3)^	-	0.08	0.39	0.87	0.39
43	2007	18-norabieta-8,11,13-triene^3)^	0.11	0.14	15.78	3.42	1.17
44	2055	abieta-8,11,13-triene	0.06	0.14	5.20	1.48	0.78
45	2083	abieta-7,13-diene^3)^	-	0.09	0.96	0.33	0.83
46	2158	neoabietadiene^3)^ + cis-abienol^3)^	0.44	0.05	0.10	0.03	0.68
47	2174	pimaral^3)^	0.05	-	0.60	0.91	0.01
48	2230	isopimaral^3)^	-	0.05	0.31	0.62	0.41
49	2247	palustral^3)^		0.17	1.54	2.59	0.83
50	2278	dehydroabietal	0.07	0.12	7.12	3.33	0.78
51	2313	abietal	0.06	0.06	0.83	0.61	0.60
52	2372	neoabietal^3)^	0.04	0.03	0.24	0.45	0.39
		Sum of minor and unidentified components	2.97	1.27	13.63	10.32	0.79
		Total	100	100	100	100	100

1) peak area percents from total eluted components on GC-MS; 2) identified by MS and retention index (RI) data from literature (R.P. Adams, 2007); 3) identification was based on MS-data only

**Table 2:** Percent (w/w %) composition^1)^ of components in fir, spruce and cedar.

Nr	RI	Compounds^2)^	*A.* *nordmanniana*	*A.* *cilicica*	*A.* *equi-trojani*	*A. bornmulleriana*	*P. orientalis*	*C. libani*
1	925	tricyclene	0.08	0.04	0.11	0.17	0.47	0.03
2	936	α-pinene	65.74	53.03	64.21	70.58	23.41	12.30
3	949	camphene	0.89	0.60	0.78	0.93	1.13	0.24
4	955	2,4 (10)-thujadiene	0.79	0.07	0.97	0.44	0.41	0.04
5	978	β-pinene	9.62	10.88	8.17	8.60	32.67	8.25
6	989	β-myrcene	0.49	21.33	0.26	2.54	2.50	4.94
7	1003	1,5,8-p-menthatriene^3)^	0.23	0.03	0.23	0.06	0.20	0.05
8	1010	3-carene	0.88	1.12	-	0.03	0.13	0.11
9	1021	p-cymene	0.36	0.15	0.54	0.34	0.45	0.46
10	1026	limonene	7.24	5.43	1.79	1.16	14.99	17.71
11	1087	α-terpinolene	0.10	0.24	0.13	0.10	0.19	0.29
12	1088	p-cymenene	0.21	0.07	0.50	0.22	0.16	0.10
13	1101	perillene	-	0.17	-	0.05	0.15	0.08
14	1123	α-campholene aldehyde	0.51	0.04	0.76	0.20	0.43	0.01
15	1130	4-acetyl-1-methylcyclohexene^3)^	0.05	0.01	-	-	0.04	0.18
16	1135	trans-pinocarveol	1.18	0.16	2.17	0.57	2.62	0.18
17	1138	cis-verbenol	0.20	0.01	0.13	-	0.07	0.01
18	1141	trans-verbenol	0.79	0.04	0.58	0.24	0.16	0.02
19	1145	p-mentha-1,5-dien-8-ol^3)^	0.45	0.04	0.75	0.18	0.28	0.01
20	1156	pinocarvone	0.21	0.06	0.35	0.07	0.75	0.04
21	1162	borneol	0.11	0.05	0.17	0.30	0.40	0.02
22	1165	p-mentha-1,5-dien-8-ol	0.99	0.03	1.23	0.25	0.72	0.03
23	1177	4-terpineol	0.07	0.08	0.14	0.11	0.29	0.14
24	1190	α-terpineol	0.47	1.17	1.52	0.75	0.77	0.33
25	1194	myrtenal + myrtenol	1.19	0.21	2.42	0.64	3.02	0.18
26	1206	verbenone	0.84	0.06	4.12	-	0.27	0.01
27	1218	trans-carveol	0.20	0.02	0.31	0.05	0.30	0.08
28	1235	thymol methyl ether	-	-	-	-	0.04	0.17
29	1242	carvone	0.08	0.02	0.11	0.02	0.24	0.08
30	1286	bornyl acetate	-	-	0.32	-	1.94	0.18
31	1374	α-copaene	-	0.02	0.10	0.13	0.91	1.17
32	1420	β-caryophyllene	0.95	0.04	0.25	0.42	1.35	0.44
33	1453	α-humulene	0.70	0.02	0.13	0.25	0.42	0.10
34	1464	β-farnesene^3)^	0.01	0.02	-	-	-	0.30
35	1478	gamma-muurolene	0.01	0.09	0.08	0.24	0.03	0.08
36	1484	germacrene-D	0.02	0.88	1.01	1.85	0.12	0.01
37	1502	α-muurolene	0.02	0.04	0.19	0.46	0.29	0.13
38	1514	gamma-cadinene	0.01	0.06	0.09	0.25	-	0.02
39	1525	trans-calamenene + Δ-cadinene	0.04	0.12	0.40	0.65	0.12	0.20
40	1546	cis-α-bisabolene^3)^	0.64	0.62	0.03	0.06	0.07	4.66
41	1578	caryophyllene oxide	0.67	-	0.21	0.26	2.16	0.18
42	1607	humulene epoxide	0.28	-	-	0.14	0.36	0.03
43	1642	α-muurolol	0.03	0.06	0.34	0.63	0.02	0.01
44	1974	manoyl oxide	0.07	0.13	0.08	0.06	0.12	0.26
45	2005	palustradiene^3)^	-	-	-	-	0.10	7.05
46	2007	18-norabieta-8,11,13-triene^3)^	0.09	0.05	0.17	0.03	0.28	-
47	2055	abieta-8,11,13-triene	0.02	0.16	0.07	0.02	0.27	17.00
48	2083	abieta-7,13-diene^3)^	0.07	0.09	0.12	0.02	1.11	8.32
49	2158	neoabietadiene ^3)^	0.03	0.03	0.04	-	0.10	0.87
50	2247	palustral^3)^	0.01	0.13	0.04	-	0.05	0.33
51	2278	dehydroabietal	0.03	0.16	0.10	0.01	0.06	0.36
52	2304	7-oxo-abieta-8,11,13-triene^3)^	-	-	-	-	-	1.07
53	2313	abietal	0.15	0.20	0.35	0.05	0.55	0.34
54	2372	neoabietal^3)^	0.03	0.05	0.07	0.01	0.55	0.08
		Sum of minor and unidentified components	2.15	1.87	3.36	5.86	2.26	10.75
		Total	100	100	100	100	100	100

1) peak area percents from total eluted components on GC-MS; 2) identified by MS and retention index (RI) data from literature (R.P. Adams, 2007); 3) identification was based on MS-data only.

**Table 3:** Terpene groups in Pinaceae family cones (%).

Species	MTHK	MT-OL	STHK	ST-OL	Diterpene	Others
*A.cilicica*	93.14	1.20	1.31	-	0.66	3.69
*A.nordmanniana*	85.72	4.10	2.80	-	0.39	6.99
*A.equi-trojani*	77.22	10.70	2.85	0.10	0.65	8.48
*A.bornmülleriana*	84.00	1.80	4.17	0.41	0.18	9.44
*P.* *orientalis*	78.40	7.55	6.16	-	1.83	6.06
*C.* *libani*	57.30	0.90	6.78	-	27.01	8.01
*P.sylvestris*	19.31	2.28	13.14	-	28.94	36.33
*P.nigra*	51.08	1.44	15.70	-	16.08	15.70
*P.halepensis*	54.50	1.70	20.82	-	4.10	18.88
*P.pinea*	82.62	0.64	1.34	-	0.67	14.73
*P.brutia*	83.94	3.53	8.11	-	0.62	3.80

MTHK: Monoterpene Hydrocarbons; MT-OL: Monoterpene alcohol; STHK: Sesquiterpene Hydrocarbons; ST-OL: Sesquiterpene alcohols.

## 3. Experimental

### 3.1. Plant Material

Eleven different coniferous cones were used in this study. Four different fir species (*A.equi-trojani, A. cilicica*, *A. nordmannia, A. bormülleriana*) and five different pine species (*P. sylvestris, P. nigra, P. halepensis, P. pinea, P. brutia*) and also *C. libani* and *P. orientalis* were collected directly from different parts of the trees. Approximately 5 kg of cones were collected for each species from their growth sites just at the time of maturity and stored in -24 ºC until the laboratory studies. Species names, sampling site, collection date, climate zone, and altitude of all specimens are listed in Table 4.

**Table 4:** Names, collection place, climate zone, date and altitude of the analysed tree species.

Species	Sampling Site	Climate Zone	Collection Date	Altitude
*A. equi-trojani*	Edremit-West Turkey	Mediterranean	October, 2007	800 m
*A. cilicica*	Adana-South Turkey	Mediterranean	May, 2007	700 m
*A. nordmanniana*	Trabzon-Northeast Turkey	Temperate	October, 2007	1,000 m
*A. bornmülleriana*	Bartin-Northwest Turkey	Temperate	October, 2007	1,100 m
*P. orientalis*	Trabzon-Northeast Turkey	Temperate	October, 2007	1,200 m
*C. libani*	Adana-South Turkey	Mediterranean	May, 2007	1,300 m
*P. halepensis*	Gokova, Mugla-West Turkey	Mediterranean	November, 2007	900 m
*P. pinea*	Bartin-Northwest Turkey	Temperate	March, 2007	600 m
*P. sylvestris*	Bartin-Northwest Turkey	Temperate	September, 2007	700 m
*P. nigra*	Bartin-Northwest Turkey	Temperate	September, 2007	750 m
*P. brutia*	Izmir-West Turkey	Mediterranean	May, 2007	850 m

### 3.2. Isolation of the Essential Oil

The essential oils of each sample were obtained by hydrodistillation with a Clevenger apparatus (ILDAM CAM Ltd. Ankara-Turkey) using 100 g of fresh cones. The oils were collected for 3 h. and dried over anhydrous sodium sulphate in a sealed vial until used [23]. The results calculated as freeze dried samples were given in mL/100 g per dry raw material and given in Figure 1 and Figure 2 [24,25].

### 3.3. Essential Oil Analysis

The GC-MS analyses for the hydrodistilled samples were performed using an HP 6890-5973 GC-MSD instrument (Agilent Technologies Canada Inc., Mississauga, ON, Canada) equipped with an HP-5 capillary column (25 m/0.25 mm i.d., 0.11 μm film thickness). Helium was used as the carrier gas at 1.0 mL/min flow rate. The column oven was temperature programmed starting from 50 ºC (0.5 min) to 250 ºC , at 4 ºC/min heating rate. After 10 min of hold time at 250 ºC the temperature program was continued at 10 ºC/min to 290 ºC for 15 min. The split-injector and MS-transfer line were held at 260 ºC and 280 ºC, respectively. The MSD was operated in electron impact ionisation mode at 70 eV electron energy [26]. Compound identifications was based on mass spectra, referring to NIST98 and WILEY275 mass spectral libraries, and also comparing measured retention index (RI) values of components with literature data [27]. The quantitative area-percent measurements were based on peak-areas from the GC-MS data. Although, some researchers [28,29] have used cluster analysis to evaluate statistical data, the preliminary studies showed that there was no statistically significant differences between extraction and injections since the material was collected at one time [30,31,32,33]. Therefore, no statistical analysis was applied in this study.

## 4. Conclusions

Comparing the essential oil yields of *Pinacea* family tree cones, pine species yielded less than fir species. However, on a volatile compound basis, pine species yielded more than fir species except for α-pinene and β-pinene. On the other hand, monoterpene hydrocarbon compounds, an important group of terpenes, were more abundant in fir species rather than in pine species.
